# A novel homozygous variant in the *ATP7B* gene in a patient with Wilson’s disease: a case report

**DOI:** 10.3389/fmed.2026.1827993

**Published:** 2026-05-04

**Authors:** Valentina E. Shavrak, Irina Z. Zhalsanova, Elizaveta A. Fonova, Daria N. Erburova, Nikita A. Kolesnikov, Sergei S. Fomenko, Valeria V. Petrova, Gulnara N. Seitova, Vadim A. Stepanov, Nikolay A. Skryabin

**Affiliations:** 1 Research Institute of Medical Genetics, Tomsk National Research Medical Center, Tomsk, Russia; 2 Biotech Campus LLC, Moscow, Russia

**Keywords:** ATP7B, clinical case, copper metabolism disorder, novel variant, Wilson’s disease

## Abstract

In this article, we describe a case of a novel variant of the *ATP7B* gene identified in a boy of Short ethnicity with Wilson’s disease (WD). Wilson’s disease is a chronic autosomal recessive disorder caused by pathogenic variants in the *ATP7B* gene. The patient’s first symptoms appeared at age 10 and included persistently elevated aspartate aminotransferase and alanine aminotransferase levels, decreased ceruloplasmin levels, diffuse liver parenchymal changes, and hepatosplenomegaly. Three months later, due to the ineffectiveness of glucocorticoid therapy, a presumptive diagnosis of Wilson’s disease was made. At age 11, the patient was admitted to the clinical department of the Research Institute of Medical Genetics. One year later, a novel variant p.(Cys69Ter) was identified in a homozygous state in exon 2 of the *ATP7B* gene.

## 1 Introduction

Wilson’s disease (WD), also known as hepatocerebral degeneration or hepatolenticular degeneration, is a chronic disorder that is inherited in an autosomal recessive manner. The disease is characterized by copper accumulation in various tissues and damage to parenchymal organs, primarily the liver and brain. WD is caused by pathogenic variants in the *ATP7B* gene that lead to disrupted excretion of excess copper into bile and impaired copper incorporation into apoceruloplasmin (1).

A diagnosis of WD is challenging due to both marked clinical heterogeneity and the abundance of rare, population-specific pathogenic variants (2, 3). In this article, we report a novel homozygous variant in the *ATP7B* gene, identified in a patient with Wilson’s disease from a Shor family. The prevalence of rare diseases often varies considerably among ethnic groups, a phenomenon that is particularly pronounced in smaller populations, where higher levels of inbreeding and the effects of genetic drift are more common. Therefore, a more comprehensive understanding of the carrier frequency of pathogenic variants within these populations can significantly improve the diagnosis of rare diseases.

## 2 Patient report

A Shor family was referred to the Genetics Clinic of the Research Institute of Medical Genetics at the Tomsk National Research Medical Center due to a suspected diagnosis of WD in their 12-year-old son.

The first symptoms appeared at 10 years and 9 months of age, reportedly after the accidental consumption of an alcoholic beverage, which triggered the development of liver failure and the subsequent manifestation of WD. The proband was admitted to the toxicology department with signs of intoxication. The initial examination revealed abnormalities in blood biochemical parameters, specifically elevated aspartate aminotransferase (AST) up to 247 U/L, alanine aminotransferase (ALT) up to 390 U/L, and alkaline phosphatase up to 815 U/L. A follow-up test 1 month later showed persistently elevated levels (AST 316 U/L; ALT 364 U/L). An abdominal ultrasound revealed diffuse changes in the liver parenchyma and hepatosplenomegaly. Following a gastroenterology consultation, a diagnosis of toxic hepatitis was made, and in accordance with clinical recommendations, glucocorticoid therapy (prednisolone 20 mg/day) was prescribed (4). To make a differential diagnosis, markers for hepatitis A, B, and C were tested, with negative results. The examination also noted decreased ceruloplasmin levels.

Three months later, a further decrease in ceruloplasmin levels was detected, along with an absence of response to the administered therapy, leading to a suspicion of Wilson’s disease. Appropriate therapy with D-penicillamine (750 mg/day) was initiated.

At 11 years and 5 months of age, the patient was hospitalized at the Clinical Department of the Research Institute of Medical Genetics. An examination was performed, revealing elevated ALT (59 U/L) and AST (52 U/L), significantly decreased serum copper (1.39 μmol/L) and ceruloplasmin (< 0.03 g/L) levels, and increased urinary copper excretion (8.16 μmol/24 h). No other diagnostic signs of WD were identified in the proband, such as Kayser-Fleischer rings, signs of central nervous system involvement, or MRI abnormalities. Coombs-negative hemolytic anemia and quantitative liver copper analysis were not performed. A molecular genetic analysis was performed to identify the common p.(His1069Gln) mutation in the *ATP7B* gene; however, this variant was not detected in the patient.

At 12 years and 5 months of age, the patient was readmitted to our Clinical Department for dynamic observation and treatment adjustments. The examination revealed elevated ALT (116 U/L) and AST (78 U/L) levels, along with decreased serum copper (1.39 μmol/L) and ceruloplasmin (< 0.03 g/L) levels, and high urinary copper excretion of 6.9 μmol/24 h. An abdominal ultrasound showed no significant structural changes. The patient and his mother were referred for whole-genome sequencing to further search for a pathogenic genetic variant of the *ATP7B* gene. According to the Leipzig diagnostic scoring system, the proband was diagnosed with WD (Table 1) (5).

**TABLE 1:** Evaluation of the proband’s symptoms using the Wilson’s disease diagnostic scoring system.

I. Typical clinical signs and symptoms of Wilson’s disease	Points	II. Other diagnostic tests	Points
1. Kayser-Fleischer rings: Absent	0	5. Quantitative liver copper analysis (in the absence of cholestasis)	–
2. Signs of CNS involvement and/or MRI abnormalities: Absent	0	6. Urinary copper excretion (in the absence of acute hepatitis): 6.9 μmol/24 h	2
3. Serum ceruloplasmin: < 0.03 g/L	2	7. Molecular genetic analysis: Homozygous	4
4. Coombs-negative hemolytic anemia	–		

Total score: 4 or more—a diagnosis of Wilson’s disease is established; 3—a diagnosis of Wilson’s disease is probable, but further patient examination is required; 2 or less—a diagnosis of Wilson’s disease is unlikely.

## 3 Results

### 3.1 Sequencing

**FIGURE 1:**
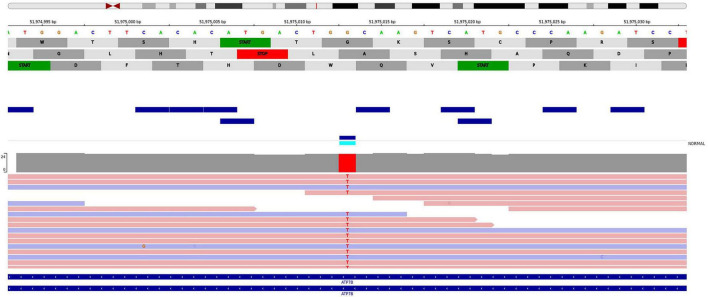
NGS sequence data of the proband (IGV browser) confirming the homozygous c.207C>A, p.(Cys69Ter) variant in the *ATP7B* gene. The *ATP7B* gene is located on the minus strand; therefore, the reference sequence shown corresponds to the positive strand, and the variant appears as G>T in this orientation, which corresponds to the c.207C>A substitution on the transcript.

**FIGURE 2:**
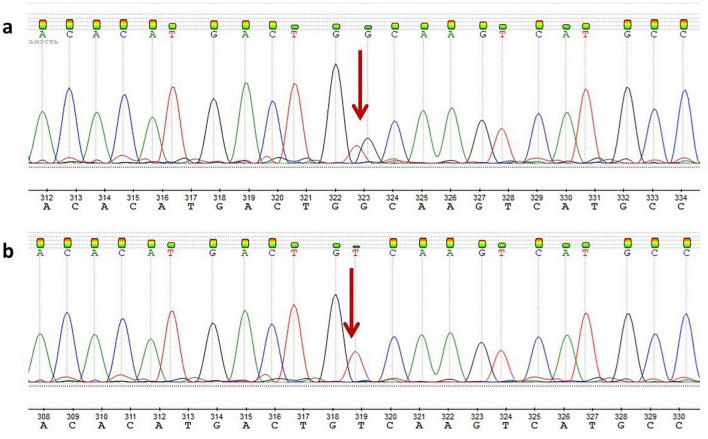
Sanger sequencing: **(a)** the proband’s mother; **(b)** the proband.

Whole-genome sequencing of the proband revealed a novel nucleotide sequence variant, c.207C>A, p.(Cys69Ter) (GRCh38:chr13:51975013G>T, NM_000053.4), in exon 2 of the *ATP7B* gene. The variant was present in a homozygous state with a read depth of 22 × (Figure 1). Sanger sequencing confirmed that the c.207C>A mutation was homozygous in the proband and heterozygous in the proband’s mother (Figure 2).

### 3.2 Pedigree of the patient

The variant was also identified in the proband’s mother in a heterozygous state. The mother denied a history of consanguinity. The proband’s father had died of causes unrelated to the disease. The proband’s asymptomatic brother and sister declined genetic testing for the variant.

### 3.3 Analysis of runs of homozygosity

Although the proband’s mother had denied consanguinity, the fact that the child was homozygous for an ultra-rare variant suggested its possible presence. To test this hypothesis, we performed a runs of homozygosity (ROH) analysis. The analysis included the following reference populations: Khakas-Sagay (*N* = 25, Tashtyp district), Khakas (*N* = 20, Askiz district), Khakas-Kachin (*N* = 19, Shirin district), Shors (*N* = 10, Mezhdurechensk and Tashtagol districts), Telengits (*N* = 24, Ulagan district), Altai-Kizhi (*N* = 93, Onguday and Ust-Koksinsky districts), Kumandins (*N* = 9, Gorno-Altaisk city and Turochak village), and Teleuts (*N* = 10, Kemerovo region).

**FIGURE 3:**
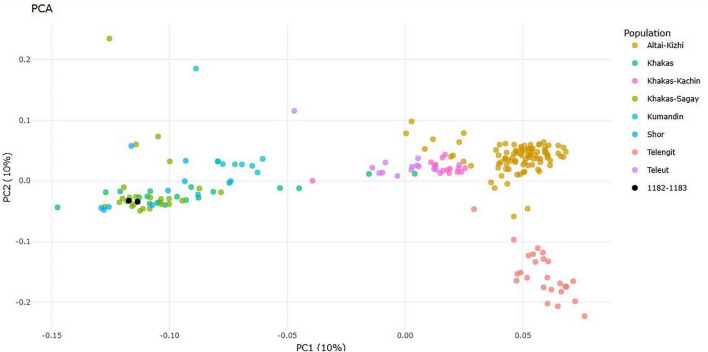
Position of clinical samples among reference populations on the PCA plot.

In the principal component analysis (PCA), the study samples cluster with the Khakas-Sagay, Khakas from the Askiz district, and Shor populations (Figure 3). A distinct group of Khakas and Shors, characterized by a low total length and number of ROH, is evident. The genomic inbreeding coefficient (FROH, calculated for ROH > 500 kb) was 0.060 for the Khakas-Sagays, 0.003 for the Shors, 0.071 for the proband, and 0.059 for the proband’s mother. These findings are consistent with their clustering in the PCA and indicate an origin in a population with a high level of endogamy, a characteristic feature of the Khakas-Sagay subpopulation from the Tashtyp and Askiz districts. The total ROH burden was 200.4 Mb for the proband and 166.7 Mb for the proband’s mother. The number of segments was nearly identical (174 for the proband and 173 for the mother). The median segment length was also very similar (661 kb for the proband and 679 kb for the mother). The length distribution of ROH segments showed a congruent profile.

## 4 Discussion

It is well-established that pathogenic variants in the *ATP7B* gene lead to impaired copper excretion into the bile and disrupted incorporation of copper into apoceruloplasmin. The *ATP7B* gene, which is responsible for WD, is located on the long arm of chromosome 13 (13q14.3). It consists of 21 exons and spans over 79 kb. Mutations in this gene cause hepatic copper overload, leading to hepatocyte death. The released copper enters the bloodstream and accumulates in extrahepatic tissues, including the brain, kidneys, and cornea (1). To date, over 900 pathogenic variants of the *ATP7B* gene have been reported. Missense mutations are the most frequent molecular cause of WD.

The most common of these mutations, including p.(His1069Gln), often do not impair the protein’s copper-binding function but rather reduce its stability, leading to rapid intracellular degradation. Mislocalization of the protein, as demonstrated in a 2012 study investigating 28 *ATP7B* variants, can also disrupt its function. However, the majority of pathogenetically significant mutations lead to a complete loss of function, i.e., an inactivation of the protein’s copper-transporting capacity of the ATP7B protein, as occurs with nonsense mutations that introduce premature stop codons (6, 7).

A likely benign synonymous substitution at position 207, c.207C>T (Variation ID: 2855901), has previously been described. This substitution changes the codon “TGC” to “TGT,” both of which encode cysteine. In contrast, the nucleotide variant identified in our proband, c.207C>A, p.(Cys69Ter), has not been reported in the gnomAD v4.1.0 or ClinVar databases. In the Database of Genetic Variant Population Frequencies of the Russian Federation, this variant is present at a frequency of 0.0000082 (8). The nucleotide variant (GRCh38:chr13:51975013G>T) introduces a premature stop codon in the *ATP7B* gene, p.(Cys69Ter). According to the ACMG guidelines, the variant was classified as likely pathogenic (criteria PVS1, PM2) (9).

The identification of this ultra-rare variant in a homozygous state can be explained by the child’s birth from a consanguineous marriage and by the population-specific frequency of this variant. Despite the mother’s denial of consanguineous marriage, we considered the possibility of such a relationship. The combination of a large number of segments (NSEG) and a moderate average length (KBAVG) is suggestive of a long-term small effective population size, potentially compounded by closer consanguineous marriages in some families. This pattern is reflected in the presence of long ROH segments in several Khakas-Sagay individuals, with total lengths of 26.5, 25.1, and 22.7 Mb.

The proband and his mother are Shors, an indigenous Turkic-speaking minority population native to the southeastern part of Western Siberia. This population exhibits a high level of inbreeding, a feature that reflects its historically small population size rather than the influence of religious or cultural customs. The mother’s and the proband’s genome-wide homozygosity is not exceptional for this group. The mother is a fairly typical representative of this population. The proband, however, likely resulted from a closer consanguineous union than the population average, which led to the merging of homozygous segments and the presence of the longest ROH in the entire sample (30.5 Mb). ROH longer than 5 Mb are indicative of recent consanguinity within the last 5-10 generations, while the presence of ROH segments greater than 10 Mb suggests consanguinity within 1-5 generations (10). The proband carries three ROH segments > 5 Mb: an 8.69 Mb segment on chromosome 1, a 5.65 Mb segment on chromosome 6, and a 30.59 Mb segment on chromosome 13. The proband’s mother carries two ROH segments > 5 Mb on chromosomes 16 and 17, measuring 5.54 and 9.91 Mb, respectively. Both the proband and his mother demonstrate a significant burden of autozygosity. The proband exhibits signs of recent consanguinity, as evidenced by the presence of several very long ROH segments. In contrast, the mother’s profile is more characteristic of the background homozygosity typical of isolated populations. This pattern can be explained by consanguineous unions within their pedigrees in the last 5-10 generations. The primary contribution to the difference in their total ROH burden is the presence of very long ROH segments in the proband. Furthermore, the fact that the mother and proband share long ROHs in different chromosomal regions indicates the presence of multiple common ancestors in their genealogies. This case is notable for the homozygous state of a rare, novel nonsense variant, which may be explained by the population-specific background of the patient.

## 5 Materials and methods

A family with a son suspected of having WD was enrolled in this study. The research was conducted in accordance with the ethical standards set forth in the World Medical Association’s Declaration of Helsinki. Informed consent was obtained from all family members prior to their participation. The study protocol was approved by the Biomedical Ethics Committee of the Research Institute of Medical Genetics at the Tomsk National Research Medical Center (registration number 173). The family’s genomic investigation was performed as part of a joint research program with the Biotech Campus LLC.

### 5.1 Whole-genome sequencing

Whole-genome sequencing was performed on the DNBSEQ-T7 sequencer (MGI) using the MGIEasy FS PCR-Free Library Prep Set (MGI). The read length was 2 × 150 bp. For each sample, the number of reads with a quality score of at least Q30 constituted no less than 90% of the total reads generated. Sequencing data processing was conducted according to the GATK best practices for “Germline SNPs and Indels” using the GATK4 software package. Read quality assessment was performed with Qualimap. Read alignment to the human reference genome (GRCh38/hg38) was carried out using BWA. Variant annotation was performed with Annovar. The clinical relevance of the identified variants was assessed using the OMIM and ExAC databases, the MutationTaster prediction tool, and a review of the existing literature.

### 5.2 Sanger sequencing

Sanger sequencing was performed to confirm the identified variant (GRCh38:chr13:51975013G>T) in the proband and his mother. The target region was amplified using the following primers: forward, 5’-GAGAAGCTGGGATGTTGTAG-3’; reverse, 5’-GGTTGCTGAGTGAGACTTTG-3’. PCR was carried out with a BioMaster HS-Taq PCR-Color kit (Biolabmix, Russia). The sequencing reaction was performed using a BrilliantDye Terminator Cycle Sequencing v3.1 kit (NimaGen, the Netherlands). Post-reaction purification was performed with the D-Pure DyeTerminator Cleanup Kit (NimaGen, the Netherlands), followed by ethanol precipitation. Sequencing was performed using a NANOFOR 05 genetic analyzer (Syntol, Russia).

### 5.3 Analysis of runs of homozygosity

We performed an analysis of ROHs using the PLINK 2.0 software. Initial data filtering involved the exclusion of single-nucleotide polymorphisms (SNPs) with a minor allele frequency of less than 5% (maf 0.05), a per-SNP missing genotype rate exceeding 10% (geno 0.1), and a per-individual missing genotype rate exceeding 10% (mind 0.1). Following quality control, 3.81 million SNPs remained for the analysis.

ROH were identified using the following parameters: a minimum physical length of 500 kb (homozyg-kb 500), a minimum of 50 SNPs per segment (homozyg-snp 50), a minimum density of one SNP per 50 kb (homozyg-density 50), and a maximum allowed gap between consecutive SNPs of 1,000 kb (homozyg-gap 1,000). The sliding window parameters were set as follows: a window size of 50 SNPs (homozyg-window-snp 50), permitting a maximum of one heterozygous call per window (homozyg-window-het 1), a maximum of five missing genotypes per window (homozyg-window-missing 5), and a threshold of 0.05 for the proportion of overlapping homozygous windows required to define an ROH (homozyg-window-threshold 0.05).

To estimate the level of genomic inbreeding, we calculated the FROH statistic, which is defined as the fraction of the autosomal genome covered by ROHs relative to their total length. This analysis was performed for ROH longer than 500 kb. The total length of the autosomal genome was assumed to be 2,800 Mb.

### 5.4 Principal component analysis

PCA was performed to determine the population origin of the clinical samples and to visualize their genetic proximity to the reference populations. The analysis was conducted using a quality-controlled dataset that excluded variants with a minor allele frequency (MAF) of less than 1%, a missing genotype fraction per variant greater than 2%, and variants deviating from Hardy-Weinberg equilibrium (*p* < 1 × 10^–10^). From the resulting dataset of 4,232,116 SNPs, 212 individuals representing the target ethnic groups in Siberia, along with two clinical samples (the proband and his mother), were selected. Principal component analysis was performed using the (pca) algorithm implemented in PLINK2. The first two principal components (PC1 and PC2) were used for visualization. Plotting and sample annotation were performed in R (v4.x.x) using the ggplot2 and plotly packages.

## 6 Conclusion

This study identified a novel likely pathogenic variant in the *ATP7B* gene (GRCh38:chr13:51975013G>T), which introduces a premature stop codon p.(Cys69Ter). A clinical and genetic diagnosis of Wilson’s disease was established for the patient. This finding will guide treatment optimization and enable preventive strategies for the patient’s family members. Further research is needed to identify other population-specific pathogenic and likely pathogenic variants. The patient’s affiliation with a local ethnic group and the identification of this previously undescribed variant in a homozygous state represent more than an isolated diagnostic finding. They indicate the existence of a broader layer of unexplored genetic variability specific to small, isolated populations. Systematic investigation of these population-specific variants is crucial to ensuring comprehensive and accurate diagnoses for all patients, irrespective of their ethnic origin. Furthermore, this investigation is essential for developing equitable and effective healthcare frameworks that account for the genetic diversity of a nation’s entire population.

## Data Availability

The original contributions presented in the study are included in the article/supplementary material, further inquiries can be directed to the corresponding author.
